# Effect of impregnation with polyethylene glycols (PEGs) of different end groups on gas separation performance of cross-linked polyethylene oxide (PEO) membranes

**DOI:** 10.1371/journal.pone.0346667

**Published:** 2026-04-10

**Authors:** Shanshan Ji, Teng Wang, Lu Guan, Chengyang Zhao, Shuai Quan, Xuewei Dong, Xianzhi Zhang, Fandi Meng, Zhicheng Tian

**Affiliations:** Department of Biological and Chemical Engineering, Jining Polytechnic, Jining, Shandong, China; Central University of Haryana, Mahendergarh, INDIA

## Abstract

Polymer membranes offer an extremely attractive solution for achieving sustainable carbon dioxide capture and have the potential for large-scale application. The authors prepared almost amorphous polyethylene oxide separation membranes through cross-linking methods, with a CO_2_/H_2_ separation coefficient of 7.9. However, there is still the drawback of low CO_2_ permeability. To improve the performance of the cross-linked membranes, this paper utilized the swelling property of the cross-linked membranes and immersed them with different end groups of PEG aqueous solutions. The effects of different end groups on the gas separation performance of the cross-linked membranes were investigated. The results showed that after being impregnated with different end group PEG, the gas permeabilities of the cross-linked membranes increased. When using a polyethylene glycol with a molecular weight of 250 g/mol for impregnation, a 47.0wt.% increase in weight was observed, and the gas permeability and CO_2_/H_2_ separation coefficient of the cross-linked membranes increased significantly. The CO_2_ permeability increased from 170 Barrer of the original membrane to 1457 Barrer, and the separation coefficient of H_2_ increased from 7.9 to 13.

## 1 Introduction

The massive emission of carbon dioxide (CO_2_) is one of the main causes of global climate change, seriously threatening the human living environment [1,2] Therefore, it is necessary to effectively capture and separate CO_2_. Compared with traditional CO_2_ separation methods, membrane technology has the advantages of good stability, high efficiency, low cost, simple operation and less environmental pollution [3–5]. Polyethylene oxide (PEO) materials are considered as one of the best materials for preparing CO_2_ capture gas separation membranes because the ether oxygen groups contained in them have a strong interaction with CO_2_, facilitating the mass transfer of CO_2_ in the membrane [6,7]. The solubility coefficient of gas in polymers is mainly affected by the compressibility of the gas itself and the interaction between the gas and the polymer matrix [8]. The ether oxygen functional groups in the PEO structure can form a strong tetrahedral dipole distance interaction with CO_2_, thus PEO materials have a high CO_2_/light gas solubility selectivity (CO_2_ is much greater than H_2_), and there is often a trade-off relationship between the solubility selectivity and diffusion selectivity in the CO_2_/H_2_ separation process (S_CO2/H2_ is high, D_CO2/H2_ < 1) [9–11]. During the separation process, solubility selectivity plays a dominant role, with CO_2_ being the fast gas and H_2_ being the slow gas, thereby achieving the reverse selection of CO_2_. However, traditional PEO materials have a high crystallinity due to their regular chain segments, which significantly reduces the gas permeability of the crystalline region [12–14]. Sun et al.[15] prepared high-performance PEO membranes (named BPM) via UV-crosslinking of bisphenol A ethoxylate diacrylate (BPA), poly(ethylene glycol) methyl ether acrylate (PEGMEA) and low molecular weight PEGDME without additional solvents. When PEGDME content reached 50 wt%, the membrane showed an unprecedented CO₂ permeability of 4883 Barrer with a high CO₂/N₂ ideal selectivity of 43, surpassing the 2019 CO₂/N₂ upper bound. This aligns with the broader goal of optimizing CO₂/N₂ separation performance, a key focus in carbon capture research [16]. Fan et al.[17] proposed a MOF-in-COF strategy for CO₂/H₂ separation: confining MOF unit cells in 2D COFs’ 1D channels to build hierarchical pores. The membranes showed ultrahigh H₂ permeance (>3000 GPU) and CO₂/H₂ selectivity exceeding Robeson upper bounds (via size sieving and fast transport), addressing pure COFs’ wide pores and traditional bilayer membranes’ underused sieving, offering a new high-performance gas separation paradigm. Therefore, preparing amorphous PEO membrane materials is very important. To inhibit the crystallization of PEO materials, scholars at home and abroad have adopted methods such as blending, copolymerization and cross-linking [18–20]. To increase the CO_2_ permeability, Castro-Munoz et al. [21] prepared Matrimid 5218/PEG200 blend membranes with different PEG contents, and conducted single gas permeation tests at 35°C and 10 bar feed pressure. The results showed that the CO_2_ permeability increased with the increase in additive content, from 7.7 Barrer of the pure membrane to 22.0 Barrer of the blend membrane with 20wt.% PEG200 content. The CO_2_/CH_4_ selectivity slightly increased from 35 of the pure membrane to 40 of the blend membrane with 5wt.% PEG200 content. When the content is higher, it decreases. In contrast, Vroulias et al.[8] observed that increasing IL loading (from 30 to 40 wt%) in PEO-based copolymer membranes consistently improved CO₂ permeability, with the 40 wt% IL-loaded membrane showing significantly higher permeability than the 30 wt% counterpart, which may be attributed to the stronger plasticization effect of higher IL content. In addition, ether groups, endowed with excellent CO₂ affinity, have also been incorporated into polynorbornene matrices for the fabrication of gas separation membrane materials. Alentiev et al. reported the synthesis and gas transport performance of two series of addition-type and metathesis polynorbornenes grafted with flexible ether moieties as pendant groups. The resulting polymers were found to be amorphous and exhibited enhanced solubility-controlled CO_2_ permeation behavior [22]. Additionally, Medentseva investigated the gas permeability of two isomeric vinyl-addition polymers synthesized from commercially available ester-functionalized norbornenes; these polymers displayed outstanding ideal and mixed-gas selectivity, along with favorable permeability for several industrially relevant gas pairs. Notably, acetoxy-functionalized polynorbornene (AcPNB) achieved a CO_2_ permeability of 270 Barrer, with a CO_2_/N_2_ separation selectivity exceeding 20.[23]

Despite this, completely amorphous PEO membranes still have problems of low gas permeability and low gas separation coefficient [24,25]. This paper represents a highly versatile strategy for markedly enhancing the gas transport performance of swellable PEO membranes. Leveraging the swelling behavior of cross-linked PEO membranes in organic solvents, we intentionally incorporated low-molecular-weight polyethylene glycol (PEG) into the PEO membrane matrix, and investigated the influence of different functional group PEG immersion on the gas permeability of the cross-linked membranes under similar molecular weight conditions. At the same time, the dissolution-diffusion theory was used for explanation.

## 2 Experiment

### 2.1 Experimental materials

O,O′-bis (2-aminopropyl) polypropylene glycol-block-polyethylene glycol-block-polypropylene glycol (PEO-600, molecular weight of 600 g/mol) and poly (ethylene glycol) diglycidyl ether (PEO-526, molecular weight of 526 g/mol) were purchased from Sigma-Aldrich and used as received. Poly(ethylene glycol) (PEG: molecular weight of 300 g/mol and 400g/mol, PEG300/PEG400), Methoxypolyethylene glycol (PEGME: molecular weight of 350 g/mol) and Poly(ethylene glycol) dimethyl ether (PEGDME: molecular weight of 250 g/mol) were purchased from Sigma-Aldrich and used as received.

### 2.2 Preparation of cross-linked PEO membranes and impregnated membranes

The preparation of cross-linked PEO membrane has been described in our previous work PEO-600 and PEO-526 were mixed directly according to the mole ratio of reactive groups with magnetic stirring under room temperature [26]. After continuously stirring more than 6 hours, the mixed homogeneous liquid was poured onto a Teflon dish. Subsequently, the membrane was pre-cured at 80°C for 3 hours. Then, the temperature was raised to 100°C with a heating rate 1°C/min and kept there for 2 hours. Finally, the temperature was raised to 120°C with a heating rate 0.5°C/min and maintained at 120°C for 0.5 hour. The obtained membranes (named CM) were ready for characterizations.

The known weight (m_1_) cross-linked PEO membranes were immersed in the PEG-water solution (70/30wt%). After 2h of ultrasonication to promote the incorporation of PEG molecule in the solution into cross-linked PEO membranes, the solution in which the membranes were immersed was standing under room temperature for 12 ~ 48h. Then, the Impregnated Membrane (IMs) were washed with distilled water immediately to remove PEG on the surface of IMs. The IMs were annealed at 60°C in vacuum for 48h and weighted as m_2_. As a result, a serial of IMs with different high loading rate ((m_2_-m_1_)/m_1_* 100%) were obtained. The IMs with different PEG loading rate were denotedas IM-x-y, where x represented PEG with different end group (EG represented PEG300 or PEG400, ME represented PEGME350 and DME represented PEGDME250), the y represented loading rate. The photographs of the membranes can be found as S1 Fig(Supporting information).

### 2.3 Measurements of pure gas transport properties

The solution-diffusion mechanism was employed to explain the gas transport properties through dense membranes. The permeability(P) with the unit of Barrer (1 Barrer = 10^−10 cm3^(STP) cm/cm^2^ sec cmHg) can be expressed as:


P=D×S (1)

where D with the unit of cm^2^/s and S with the unit of cm^3^(STP)/cm^3^ polymer cmHg are the diffusivity and solubility coefficients, respectively. The ideal selectivity of a membrane for gas A over gas B can be defined as follows:


$$\alpha_{\frac{A}{B}}=\frac{P_{A}}{P_{B}}=\left(\frac{D_{A}}{D_{B}}\right)\times \left(\frac{S_{A}}{S_{B}}\right)$$ (2)

The pure gas permeability was obtained by using a constant volume method. The permeabilities of various gases were tested in the sequence of H_2_, N_2_ and CO_2_. The gas permeabilities were measured at 35°C. The H_2_ is tested under 3.5 atm for safety reason and other gases are tested under 10 atm. For comparisons, CO_2_ gas is tested under both 10 atm and 3.5 atm. The gas permeability was determined from the rate of downstream-pressure increase (d*p*/d*t*) obtained when permeation reached steady state according to the following equation:


$$P=\frac{\text{2}\text{7}\text{3}\times {\text{1}\text{0}}^{\text{1}\text{0}}}{\text{7}\text{6}\text{0}}\frac{Vl}{AT\left(p_{\text{2}}\times \frac{\text{7}\text{6}}{\text{1}\text{4}.\text{7}}\right)}\left(\frac{dp}{dt}\right)$$ (3)

where P is the permeability of a membrane to a gas and its unit is in Barrer. V is the volume of the downstream chamber (cm^3^), l is the membrane thickness (cm). A refers to the effective area of the membrane (cm^2^), T is the testing temperature (K) and *p*_*2*_ means the upstream operating pressure (psia). The diffusivity can be calculated by the time-lag method as Equation 4 expressed, and then the solubility can be simply deduced according to Equation 1.


$$D=\frac{l}{\text{6}\theta }$$ (4)

where *θ* is the diffusion time lag extrapolated from the plot of pressure with time at steady state to the time axis.

## Results and discussion

**Fig 1:**
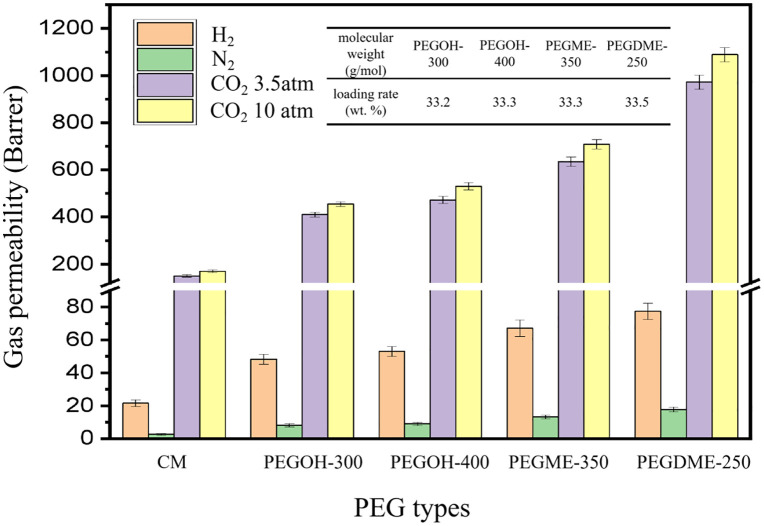
Gas permeability of IMs with different end groups.

The structure of cross-linked membranes (CM) have been described in our previous works [26], which will not be discussed here. Fig 1 shows the gas permeabilities of original membranes and IMs with different end groups.

As can be seen from Fig 1, CO_2_ permeability of all membranes are much more hihger than that of N_2_ and H_2_, which due to the strong interaction between CO_2_ and EO groups in PEO membranes causing high solubility. Interestingly, H_2_ permeability is higher than N_2_ permeability, which can be attributed to the high diffusivity caused by its smaller kinetic diameter. Meanwhile, the gas permeability of IMs impregnated with PEG solution has significantly increased compared to the original membranes. For example, the CO_2_ (10 atm) permeability increased from 170 Barrer of the original membrane to 530 Barrer of the PEGOH-400 membrane and 710 Barrer of the PEGME-350 membrane. More delightfully, the gas permeability of the membrane impregnated with PEGDME-250 was further significantly enhanced, the highest CO_2_ permeability reached 1090 Barrer. Through the above analysis, it can be found that the different end groups have a significant impact on the gas permeability of the IMs. This may be because when the PEG end group is a hydroxyl group, the small molecules PEG impregnated into the membrane are more likely to form hydrogen bonds with the cross-linked membrane segments, hindering the diffusion process of gases. When using PEGME-350, the number of hydroxyl groups decreases, and the hindering effect of hydrogen bonds weakens; when using PEGDME-250, the hydrogen bond effect is further weakened, thereby significantly increasing the gas permeability. Moreover, it can also be seen that the permeabilityes of CO_2_ and N_2_ increase significantly, while the permeability of H_2_ increases only slightly. This phenomenon indicetaes that the impregnation treatment effectively improves the free volumes of IMs which is beneficial to the gas molecules diffusion process [27,28]. However, the improved free volumes is usefulness for H_2_ due to its smaller kinetic diameter. In order to nvestigate the effect of PEG with different terminal groups on the free volume of the membrane, The positron annihilation lifetime spectroscopy (PALS) was used to determine the size of free volume and the fractional free volume of the dense membranes. A detailed description of the PALS test method can be found in the supporting information. Table 1 shows the o-Ps lifetime τ_3_, intensity I_3_ and FFV results of original membranes and IMs with different end groups.

**Table 1:** The PALS data of original membrane and FIMs.

	*τ*_*33*_ (ns)	*I*_*33*_ (%)	FFV (%)
Original membrane	2.405 ± 0.011	19.72 ± 0.12	4.867 ± 0.062
PEGOH-300	2.406 ± 0.011	20.50 ± 0.12	5.089 ± 0.063
PEGOH-400	2.415 ± 0.011	20.46 ± 0.12	5.097 ± 0.063
PEGME-350	2.442 ± 0.011	21.00 ± 0.12	5.345 ± 0.065
PEGDME-250	2.430 ± 0.011	20.54 ± 0.12	5.177 ± 0.064

According to Table 1, it appears that the τ_3_ values, which represent the mean radii of the free volume cavities according to the Tao-Eldrup model in ESI, show an increasing trend as the chang of diffrent end groups. In addition, the FFV, associated with both τ_3_ and I_3_, shows a monotonically increment, causing the significant increase of gas permeability. This indicates that that different types of end groups exert a significant influence on the FFV of the membrane. A higher FFV enables gases to diffuse more easily inside the membrane, which is also the reason for the increase in gas permeability.

**Fig 2:**
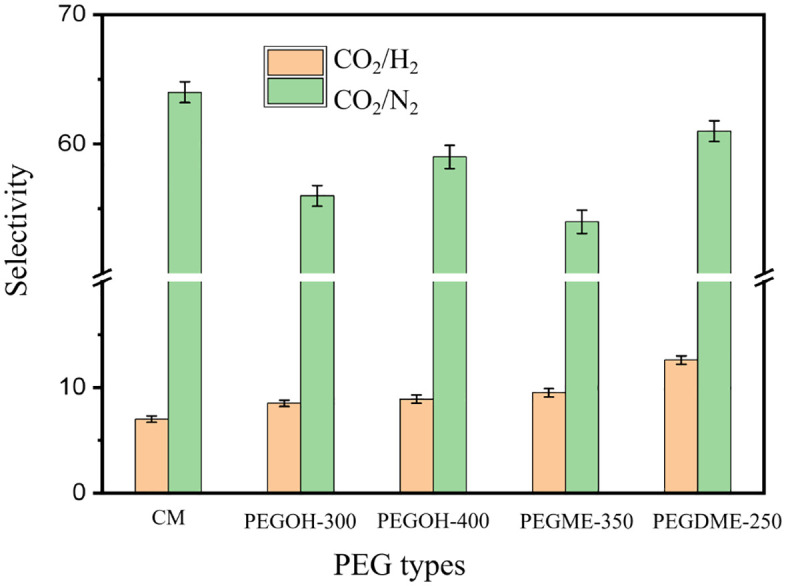
Gas selectivity of IM with different end groups.

Fig 2 shows the gas selectivities of original membranes and IMs with different end groups. From the figure, it can be seen that the CO_2_/N_2_ selectivity changes slightly, remaining between 54 and 61; while the CO_2_/H_2_ selectivity has significantly increased, from 8.5 of IM-EG300 to 12.6 of IM-DME250, approximately increasing by 50%. This is mainly due to the fact that the increase in CO_2_ permeability is greater than that of H_2_. The molecular kinetic diameter of H_2_ (2.89 Å) is much smaller than that of CO_2_ (3.30 Å) and N_2_ (3.64 Å), so the free volume change and hydrogen bond effect of the impregnated membrane have a relatively small impact on the diffusion process of H_2_, while they have a greater impact on CO_2_ and N_2_ with larger molecular kinetic diameters. Therefore, when impregnated with PEGDME-250, the permeabilities of CO_2_ and N_2_ have significantly increased due to the improvement of the diffusion process, while the permeability of H_2_ has increased slightly.

**Fig 3:**
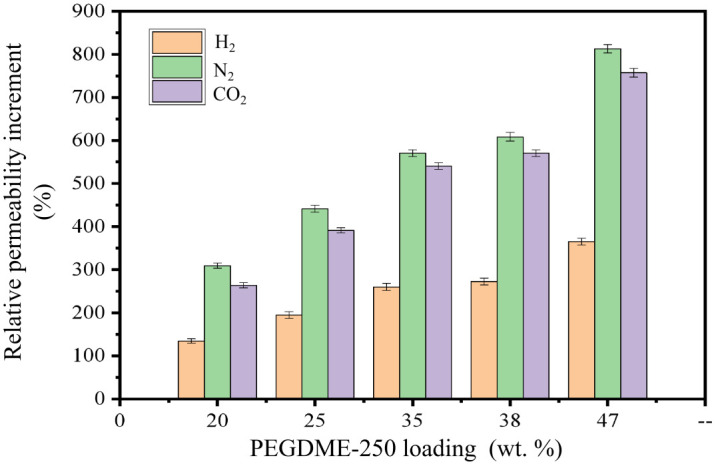
Relative permeability increment of IM-PEG.

Fig 3 shows the relative permeability increment of IM-PEGDME as a function of PEG loading compared with originan membrane. As can be seen from Fig 3, the gas permeability of IM-DME significantly increased with the increase of PEGDME250 loading rate. Moreover, the growth rates of the permeabilityes of the three test gases were in the order of N_2_ > CO_2_ > H_2_. The permeabilityes of H_2_, N_2_, and CO_2_ increased from 21.5 Barrer, 2.64 Barrer, and 170 Barrer of the original membrane to 100 Barrer, 24.1 Barrer, and 1457 Barrer with a loading rate 47.0% of IM. The growth rates of the permeabilityes reached 365%, 810%, and 750% respectively. The significant increase in gas permeability can be attributed to the free volume fraction of the membrane increased after impregnation, which is beneficial for the diffusion of gas molecules in the membrane; and after impregnation, due to the presence of PEGDME, the number of ether oxygen groups on the membrane surface increased, which can improve the dissolution process of gases on the membrane surface.Table 2 shows the o-Ps lifetime τ_3_, intensity I_3_ and FFV results of IM-PEGDME as a function of PEG loading.

**Table 2:** The FFV results of IM-PEGDME as a function of PEG loading.

PEGDME250 loading	*τ*_*33*_ (ns)	*I*_*33*_ (%)	FFV (%)
Original membrane	2.405 ± 0.011	19.72 ± 0.12	4.867 ± 0.062
19.4	2.430 ± 0.011	20.54 ± 0.12	5.177 ± 0.064
25.8	2.4242 ± 0.011	20.3069 ± 0.12	5.2564 ± 0.064
33.5	2.454 ± 0.011	21.06 ± 0.12	5.402 ± 0.065
37.5	2.475 ± 0.011	20.83 ± 0.12	5.425 ± 0.064
47	2.463 ± 0.011	21.52 ± 0.12	5.556 ± 0.065

According to Table 2, the τ_3_ values show an increasing trend as the PEGDME250 loading increases from 0 to 47.0%. This is ascribed to the increased interchain spacing arising from the incorporation of PEGDME molecules after the swelling immersion treatment. Additional free volume is created within the membrane by the embedded PEGDME molecules. In addition, the FFV shows a monotonically increment as the PEGDME loading increases, indicating that the incorporation of PEGDME molecules would create additional free volume cavities in the polymer matrix.

**Fig 4:**
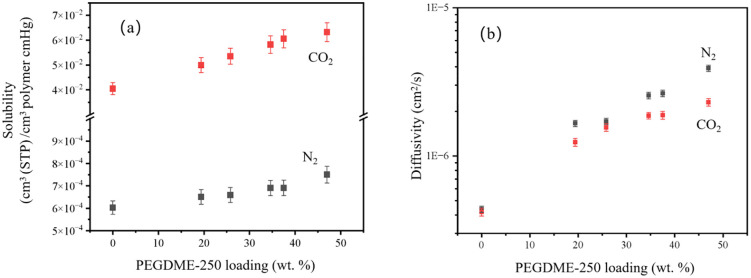
Solubility (a) and diffusivity (b) of IM-PEGDME with different loading.

Fig 4 illustrates the solubility (a) and diffusion (b) of the IM-DME with different loading rate e in which there is no data of H_2_ because the time lag of H_2_ is extremely small. The CO_2_ solubility is much more higher than that of N_2_, due to the strong interaction between CO_2_ and PEG. Meanwhile, the CO_2_ solubility inceases with the inceasing PEGDME loading rate. On the contrary, the N_2_ diffusivity is slightly higher than CO_2_ diffusivity duing to the smaller kinetic diameter of N_2_. Furthermore, the diffusivities of N_2_ and CO_2_ increse with the incresing PEGDME loading rate, attributed to the improvment of free volumes of IM-DME.

**Fig 5:**
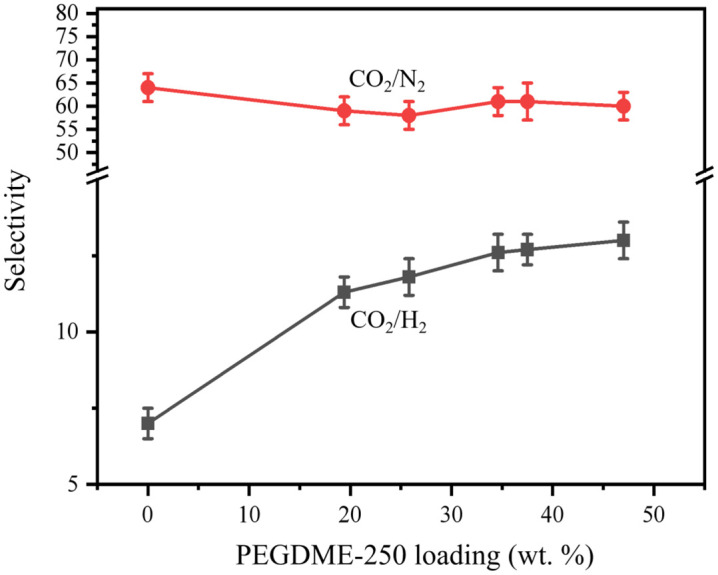
Gas selectivity of IM-PEGDME as a function of PEG loading.

Fig 5 shows the gas selectivity of IM-DME as a function of PEG loading rate. From the figure, it can be seen that the selectivity of CO_2_/N_2_ slightly decreases, from 64 of the original membrane to around 60. This can be attributed to the fact that after PEGDME impregnation, the increase in N_2_ permeability is greater than that of CO_2_. At the same time, the selectivity of CO_2_/H_2_ shows a significant increase, from 7.0 of the original membrane to 13 when the loading rate of PEGDME reaches 47.0wt.%, with an growth rate of 86%. It can be seen that the use of PEGDME-250 impregnated CM membrane has a very obvious effect on improving the gas permeability of the membrane and the separation coefficient of CO_2_/H_2_.

**Fig 6:**
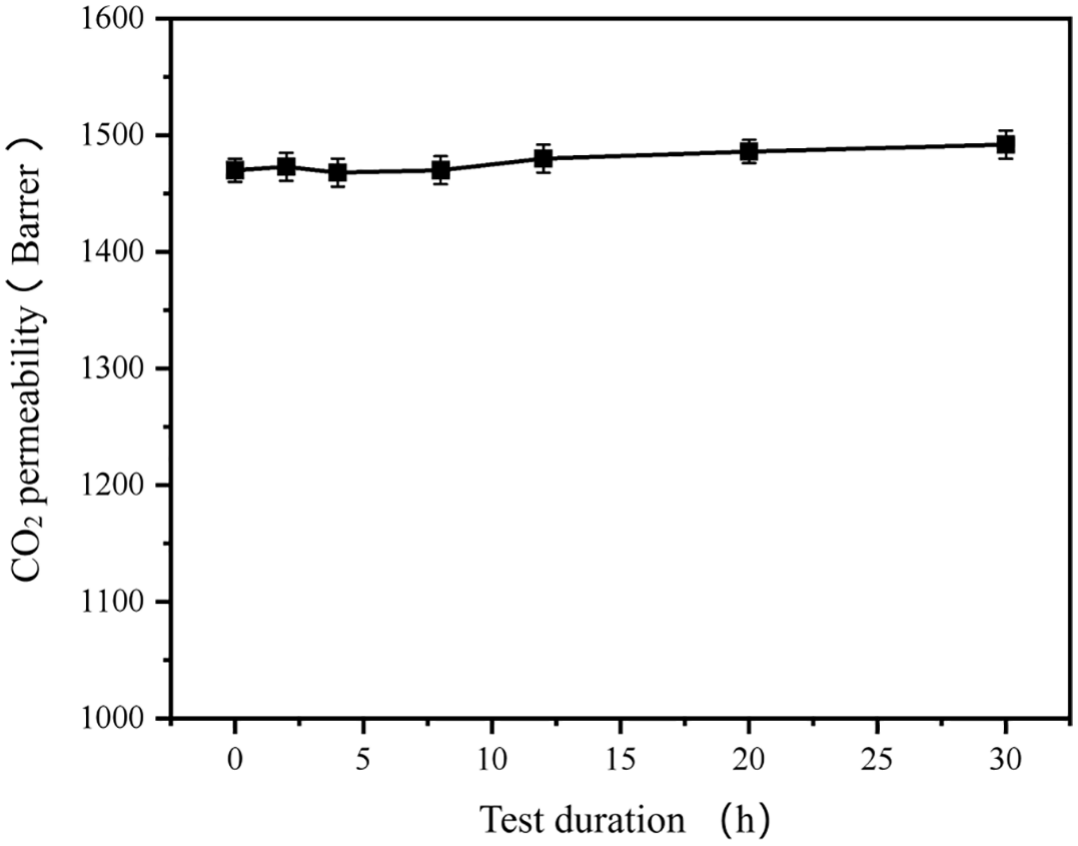
CO2 permeability of IM-DME-47.0% as a function of test duration.

In addition, time-dependent permeation performance of IMs was also investigated. Fig 6 shows the CO2 permeability of IM-DME with a loading 47.0% as a function of test duration.

According Fig 6, the CO_2_ permeability of the membrane remained relatively stable and essentially unchanged with the extension of the test time. This indicates thatthe PEG molecules are firmly entrapped within the membrane due to the cross-linked structure inside the membrane, with no leaching observed.

## Conclusions

Through this work, it was discovered that by immersing the prepared cross-linked PEO in a small-molecular-weight PEG solution, the gas permeability of the cross-linked membrane could be effectively improved. This was because the immersion treatment improved the intrinsic volume of the cross-linked membrane. Different end groups of PEG also played an important role in improving the performance of the cross-linked membrane. The results showed that the immersion treatment with dimethyl ether polyethylene glycol (PEGDME) was the most effective. Moreover, as the weight gain during immersion increased, the permeability of the cross-linked membrane also improved significantly. In this work, when the weight gain of PEGDME was 47.0 wt.%, the gas permeability of the cross-linked membrane was the best. The CO_2_ permeability increased from 170 Barrer of the original membrane to 1457 Barrer, and the separation coefficient for H_2_ increased from 7.9 to 13. This was mainly because the free volume fraction of the membrane increased after immersion, which was beneficial for the diffusion of gas molecules within the membrane; at the same time, due to the presence of PEGDME after immersion, the number of ether oxygen groups on the membrane surface increased, which improved the dissolution process of gas on the membrane surface. Meanwhile, the impregnated membranes exhibit excellent temporal stability.

## Supporting information

S1 Fig The photographs of the membranes. (DOCX)
