# Supplementary material for: Effect of impregnation with polyethylene glycols (PEGs) of different end groups on gas separation performance of cross-linked polyethylene oxide (PEO) membranes
Source: PLoS One. 2026 Apr 10;21(4):e0346667. doi: 10.1371/journal.pone.0346667 (PMC13068244; doi:10.1371/journal.pone.0346667)
Supplement: S1 Fig — (DOCX) [file pone.0346667.s001.docx]

**Effect of impregnation with polyethylene glycols (PEGs) of different end groups on gas separation performance of cross-linked polyethylene oxide (PEO) membranes**

Shanshan Ji, Teng Wang, Lu Guan, Chengyang Zhao, Shuai Quan, Xuewei Dong, Xianzhi Zhang, Fandi Meng, Zhicheng Tian

**ESI Table of Contents**

S1 Fig. The photographs of the membranes

S2 Characterizations

S1 The photographs of the membranes


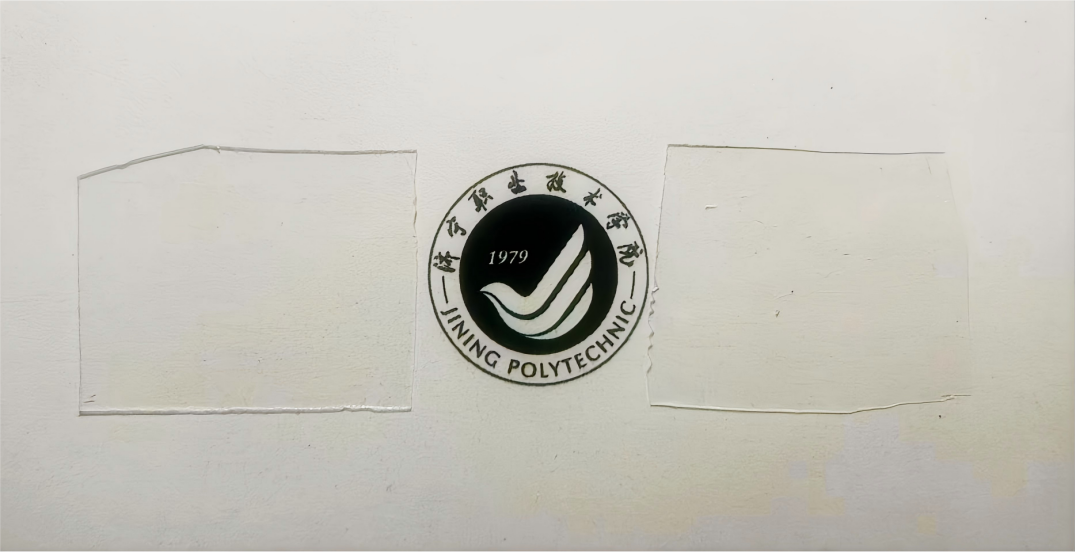


IMDME-40.7%

Original membrane

Fig S1 The photographs of the membranes

S2 Characterizations

The size of free volume and the fractional free volume of the dense films were determined by positron annihilation lifetime spectroscopy (PALS). In addition, the *Ortho*-positronium (*o*-Ps) lifetime and intensity can be related with the size and concentration of free volume in membranes. A fast-fast coincident PALS spectrometer with a system channel width of 50.53 picoseconds per channel was used to measure the lifetime and intensity of positron species. A detailed description of the experimental process can be found elsewhere. [1,2] A ^22^Na isotope sealed in Kapton film was used as positron and *γ*-ray (1274 KeV) sources. Membrane samples were cut into pieces with a dimension of 1×1 cm. The sealed source was sandwiched in between two stacks of membrane samples of about 0.5 mm thick at each side. All the measurements were performed at a counting rate of approximately 200 cps and the total number of counts for each spectrum was 1.0 million.

The PALS spectra were best resolved into three lifetime components using a PATFIT program. The para-positronium (p-Ps) lifetime, *τ_1_*, was fixed to 0.125 ns. The free positron lifetime, *τ_2_*, and the ortho-positronium (o-Ps) lifetime, *τ_3_*, with their respective intensity, *I_2_* and *I_3_*, were simulated. The o-Ps lifetime, derived from the so-called pick-off annihilation with electrons in molecules, were commonly associated with the mean radii *R* (Å to nm) of the free volume elements based on a semiempirical correlation equation derived from a Tao-Eldrup model: [3-5]
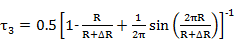

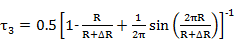

$$\tau_{3}=0.5\left[1\text{-}\frac{R}{R+\Delta R}+\frac{1}{2\pi}\sin\left(\frac{2\pi R}{R+\Delta R}\right)\right]^{-1}$$

where *R* is the free volume radius and *ΔR* is a homogeneous electron layer in the infinite potential barrier (= 1.66 Å). The relative fractional free volume (*FFV*) is calculated based on the Williams−Landel−Ferry (WLF) equation: [6,7]

[1]. N. L. Le, Y. P. Tang, T. S. Chung, The development of high-performance 6FDA-NDA/DABA/POSS/Ultems dual-layer hollow fibers for ethanol dehydration via pervaporation. J. Membrane Sci.. 2013, 447, 163-176.

[2]. Y.P. Tang, H. Wang, T.S. Chung, Towards high water permeability in triazine-framework-based microporous membranes for dehydration of ethanol, ChemSusChem, 2014, doi: 10.1002/cssc.201402816.

[3] Y.C. Jean, Q. Deng, T.T. Nguyen, Free-volume hole properties in thermosetting plastics probed by positron annihilation spectroscopy: Chain extension chemistry, Macromolecules, 28 (1995) 8840-8844.

[4] Y.C. Jean, J.P. Yuan, J. Liu, Q. Deng, H.J. Yang, Correlations Between Gas Permeation And Free-Volume Hole Properties Probed by Positron-Annihilation Spectroscopy, J. Polym. Sci. Poly. Phys., 33 (1995) 2365-2371.

[5] Y.J. Fu, J.T. Chen, C.C. Chen, K.S. Liao, C.C. Hu, K.R. Lee, J.Y. Lai, Characterization of morphology and gas separation performance of dry-cast polycarbonate membranes, Polym. Eng. Sci., 53 (2013) 1623-1630.

[6] W.J. Koros, W.C. Madden, Transport Properties, John Wiley & Sons, Inc.: New York, (2002).

[7] Y. Liu, R. Wang, T.S. Chung, Chemical cross-linking modification of polyimide membranes for gas separation, J. Membrane Sci., 189 (2001) 231-239.
